# Afatinib combined with anlotinib in the treatment of lung adenocarcinoma patient with novel HER2 mutation: a case report and review of the literature

**DOI:** 10.1186/s12957-021-02444-7

**Published:** 2021-11-18

**Authors:** Huanhuan Xu, Qi Liang, Xian Xu, Shanyue Tan, Sumeng Wang, Yiqian Liu, Lingxiang Liu

**Affiliations:** Department of Oncology, The First Affiliated Hospital of Nanjing Medical University, 300 Guangzhou Road, Nanjing, 210029 Jiangsu People’s Republic of China

**Keywords:** Afatinib, Anlotinib, HER2 mutation, Lung adenocarcinoma, Targeted therapy

## Abstract

**Background:**

HER2 is a member of the ERBB family of receptor tyrosine kinases, and HER2 mutations occur in 1–4% of non-small cell lung cancer (NSCLC) as an oncogenic driver mutation. We found a rare mutation of HER2 p.Asp769Tyr in NSCLC.

**Case presentation:**

We presented a case of a 68-year-old nonsmoking male patient with brain metastasis from lung adenocarcinoma harboring a rare mutation of HER2 p.Asp769Tyr. After multiple lines of treatment, he obtained a durable response (10 months) to afatinib and anlotinib.

**Conclusion:**

We reported for the first time that afatinib and anlotinib have successfully treated lung adenocarcinoma with HER2 p.Asp769Tyr mutation. This finding can provide an insight into the optimal treatment of lung adenocarcinoma patients with novel mutations. Additionally, we summarized the efficacy of targeted therapy for HER2 mutant lung cancer in this article.

**Supplementary Information:**

The online version contains supplementary material available at 10.1186/s12957-021-02444-7.

## Background

Human epidermal growth factor 2 (HER2, also known as ERBB2, NEU, or EGFR2) is a member of the ERBB family of receptor tyrosine kinases [1]. The ERBB2 gene, which encodes HER2, is located on the long arm of chromosome 17 (17 q21) and activates downstream signaling pathways, such as PI3K-AKT and MEK-EPK, causing cell proliferation and migration [2, 3]. HER2 has no known ligand and can be activated by homodimerization or heterodimerization with other ERBB family members. The main types of HER2 mutations include protein overexpression, gene amplification, intraframe insertion of exon 20, and point mutations [4]. HER2 mutations occur in approximately 1 to 4% of non-small cell lung cancer (NSCLC) [5]. Nonsmoking females with adenocarcinoma are more common in these patients [4].

Afatinib is an oral HER family blocker that can covalently bind and irreversibly block HER receptor family members [6]; furthermore, afatinib shows promising results and manageable toxic characteristics by targeting exon 20 mutations in NSCLC [4, 7]. Angiogenesis is essential for the occurrence and development of tumors. Anlotinib is a new oral multitargeted antiangiogenic tyrosine kinase inhibitor (TKI) that can present an inhibitory effect on tumor growth and angiogenesis by inhibiting multiple targets, such as VEGFR, PDGFR, FGFR, and C-Kit [8]. Clinically, anlotinib is an effective posterior line treatment in advanced NSCLC [9]. However, to date, there is still no relevant report about the combination of the two drugs.

HER2 p.Asp769Tyr is a novel mutation in NSCLC, and no effective clinical drug has been reported to treat this mutation. Herein, we report for the first time that afatinib combined with anlotinib targeted  HER2 p.Asp769Tyr mutation produced a durable response (10 months) after multiple lines of treatment.

## Case presentation

**Fig. 1:**
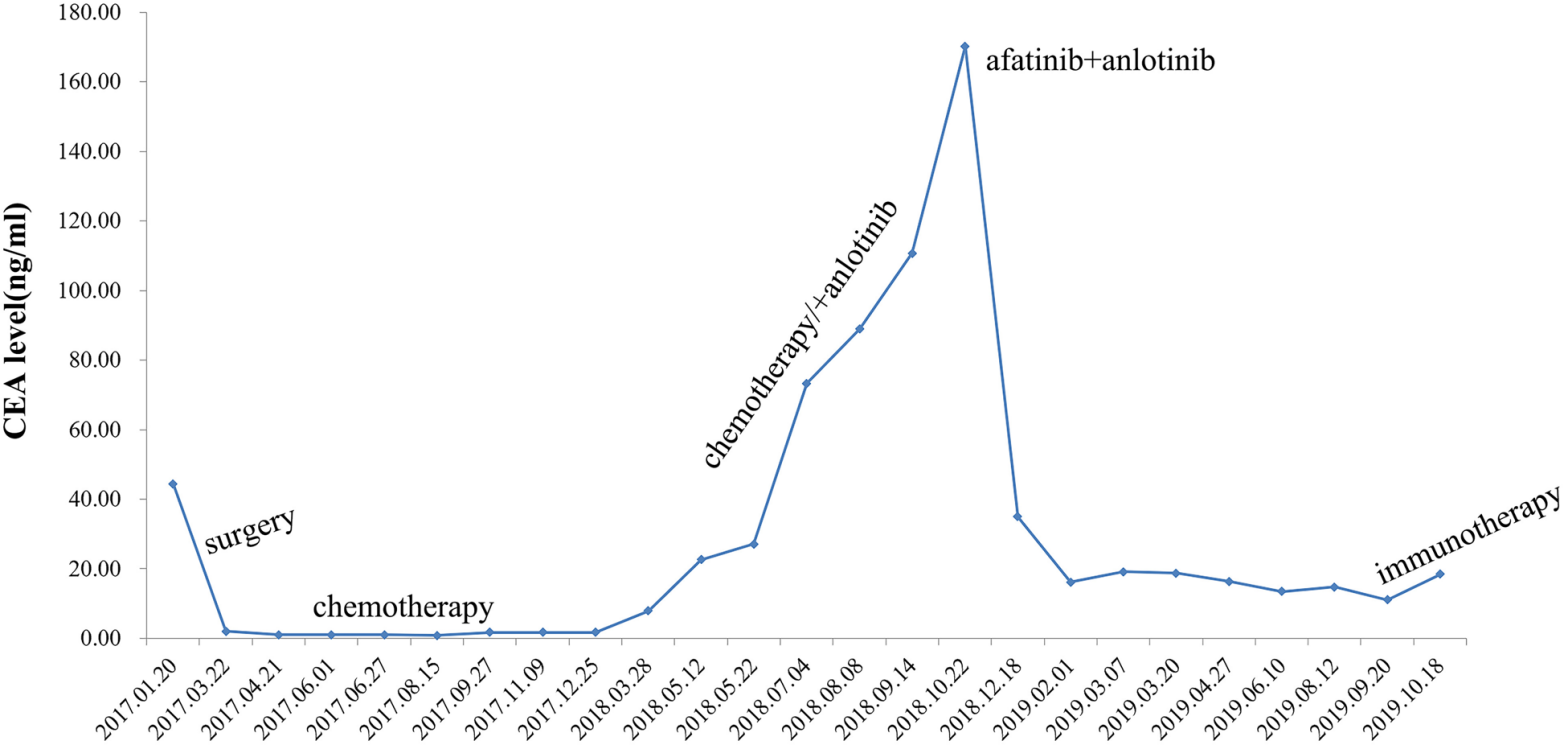
Timeline of treatment and trend in CEA level during treatment

**Fig. 2:**
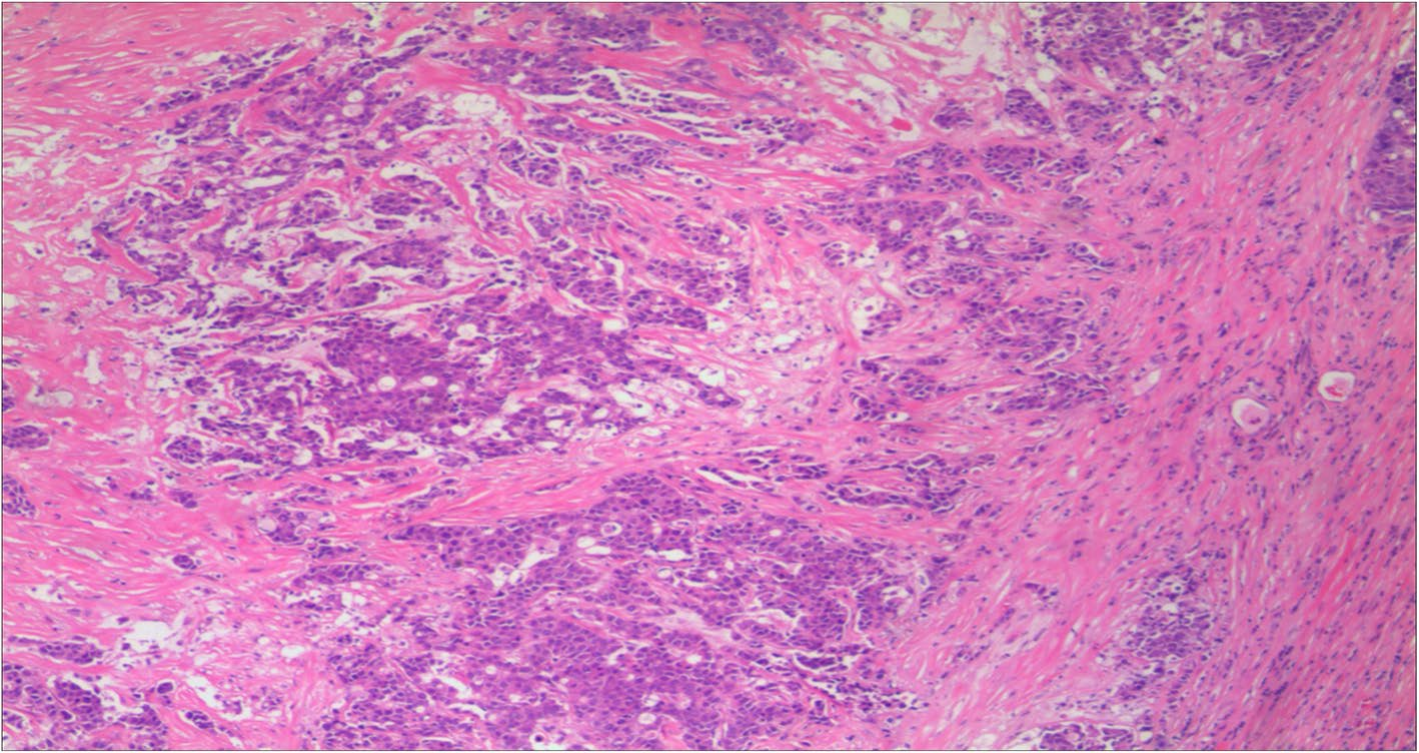
The pathological diagnosis of the resected specimens was lung adenocarcinoma (× 100)

A 68-year-old nonsmoking male was admitted to the local hospital due to elevated CEA in December 2016 (Fig. 1). An enhanced computed tomography (CT) scan of the chest and abdomen revealed a space-occupying lesion in the right upper hilum of the lung. After excluding surgical contraindications, radical resection of lung cancer plus pulmonary angioplasty was performed in our hospital on February 10, 2017. Postoperative pathology showed poorly differentiated carcinoma (Fig. 2). An immunohistochemistry (IHC) examination revealed that cytokeratin (CK)-7, CK-pan, CK-L, and CK-H were positive, while thyroid transcription factor-1 (TTF-1), CK5/6, P40, and P63 were negative. Combined with hematoxylin and eosin (HE) staining, these results were consistent with poorly differentiated adenocarcinoma. The tumor node metastasis (TNM) classification of this patient was T2aN1M0. Brain magnetic resonance imaging (MRI) showed no intracranial metastasis.

**Fig. 3:**
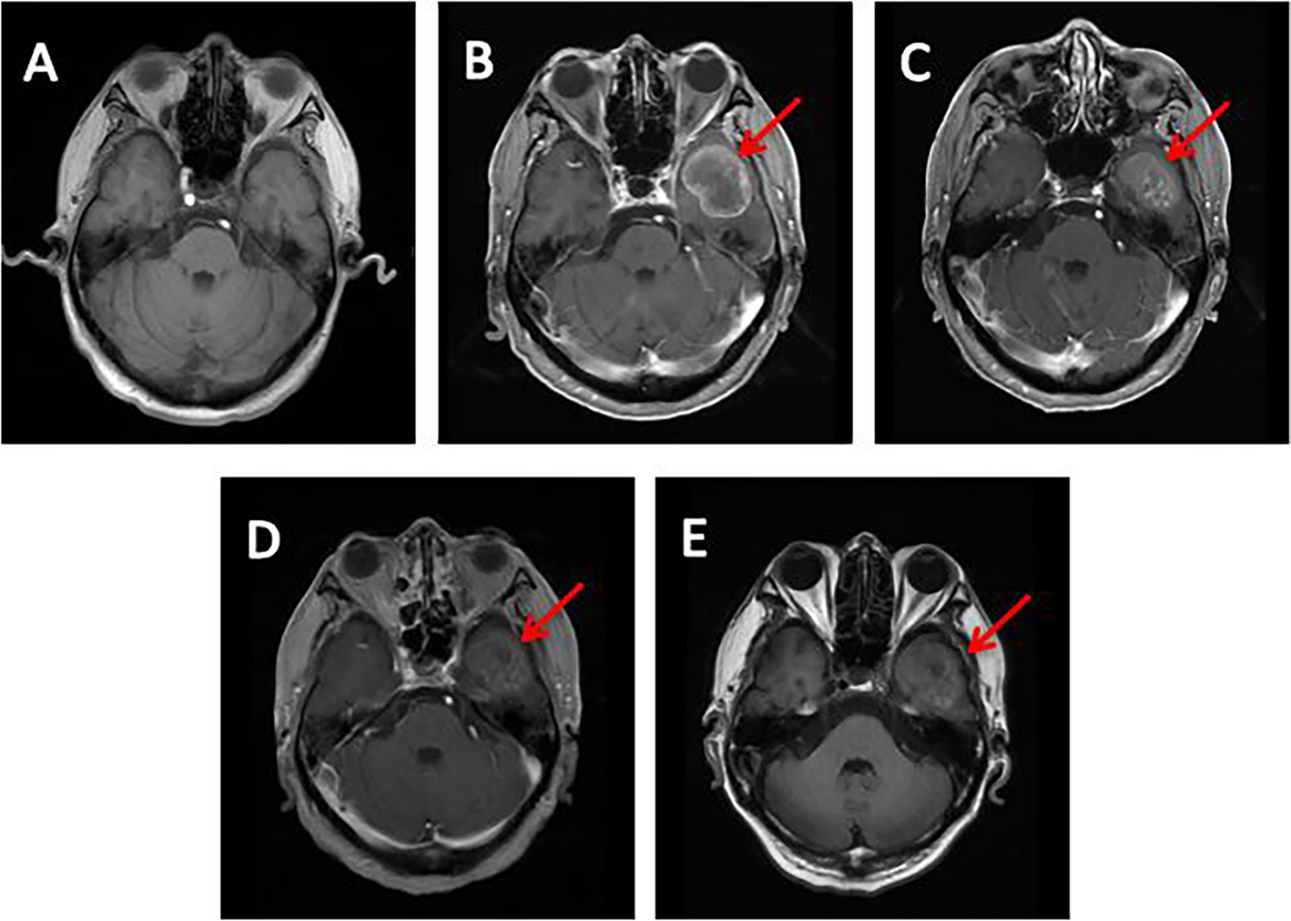
Brain magnetic resonance imaging (MRI) after treatment with afatinib and anlotinib. **A** Before any treatment. **B** Before afatinib treatment. **C** Brain MRI showing partial response after 1 month of afatinib and anlotinib. **D** Brain MRI showing stable disease after 5 months of afatinib and anlotinib. **E** Brain MRI showing progressive disease after 10 months of afatinib and anlotinib

**Fig. 4:**
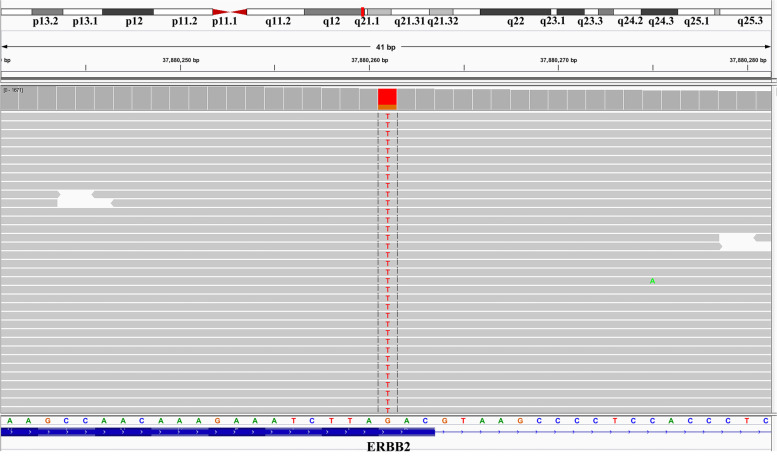
The Integrative Genomic Viewer screenshot revealed the harboring of HER2 p.Asp769Tyr

This patient received adjuvant chemotherapy with pemetrexed (500 mg/m^2^, D1) and carboplatin (AUC = 5, D2) for 4 cycles, followed by pemetrexed (500 mg/m^2^, D1) alone for 2 cycles after the operation. The last chemotherapy treatment was on September 28, 2017. The patient remained tumor-free until May 2018. He presented a gradual increase in CEA, but the CT scan was similar to before. After clinical evaluation, the patient received pemetrexed and carboplatin again. Then, based on the unsatisfactory therapeutic effect, the treatment regimen was changed to docetaxel and tegafur (50 mg/bid, d1-d14). Soon, the regimen was replaced by docetaxel and anlotinib (12 mg/day, d1-d14). Due to severe acid reflux and nausea, the patient was unable to continue taking tegafur. After 2 cycles, the CT scan still showed no recurrence or metastasis, and brain MRI revealed that the left frontal lobe and temporal lobe had round-like enhancement lesions with the sizes of 2.3 × 2.1 cm and 3.8 × 3.3 cm, respectively, and metastasis was considered (Fig. 3B). Next, the patient had received gamma knife stereotactic radiotherapy to the brain. However, the level of CEA continued to rise (Fig. 1). To establish an effective treatment, next-generation sequencing (NGS) was used for DNA sequencing analysis. The result showed that the patient had a HER2 p.Asp769Tyr mutation (c.2305G>T) (Fig. 4). Simultaneously, he also had CDKN2A, NF1, RB1, TP53 gene mutations (Supplementary Figures 1, 2, 3, 4), and HER2 gene amplification. In addition, PD-L1 expression was negative. Therefore, afatinib (40 mg/day) combined with anlotinib (12 mg/day, d1-d14) administration began on November 20, 2018, after the patient showed no clinical response to multiple prior lines of therapy. One month after receiving afatinib and anlotinib, the intracranial lesions achieved partial response (PR) according to the Response Evaluation Criteria in Solid Tumors guidelines (version 1.1) (Fig. 3C). At the same time, the level of CEA declined rapidly (Fig. 1). Then, he was followed up at outpatient visits every 2 months and achieved persistent stable disease (SD) until September 20, 2019. After taking afatinib and anlotinib for 10 months, brain MRI showed progressive disease (PD) (Fig. 3E). He gained a progression-free survival (PFS) of 10 months. The main treatment-related side effect observed was a diarrhea adverse event, but it was manageable.

Although the patient underwent serum NGS again, unfortunately, no target was observed. The patient was treated with toripalimab, pemetrexed, and anlotinib until June 17, 2020. Sadly, his brain MRI soon showed intracranial metastases with hemorrhage, and the patient passed away 2 months later.

## Discussion and conclusions

**Fig. 5:**
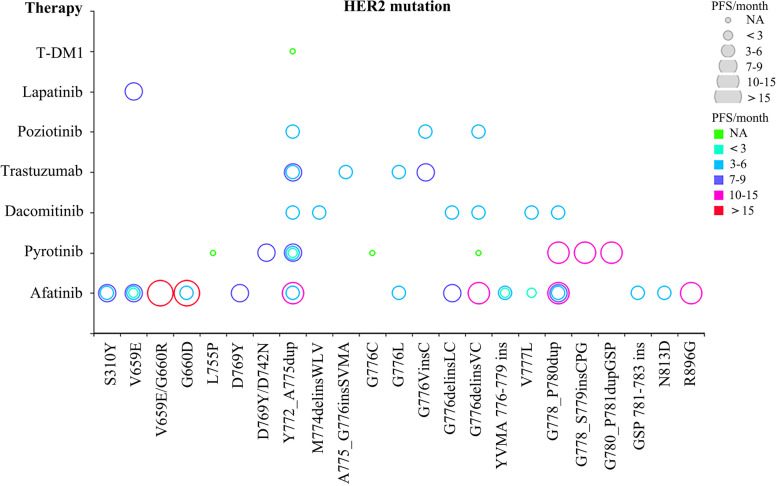
The location of HER2 mutations and corresponding targeted therapies in NSCLC, as well as the progression-free survival after treatment. NA means to have a clinical effect but no specific PFS. PFS refers to the mPFS in dacomitinib treatment of HER2 mutations

Lung cancer is the most common cause of cancer-related deaths worldwide. NSCLC is the main form of lung cancer, accounting for 85% of lung cancer patients. At present, there are various treatments for NSCLC, including chemotherapy, immunotherapy, and targeted therapy [10]. The vast majority (92%) of HER2 mutations were in-frame insertions in exon 20 (from 3 to 12 base pairs) between codons 775 and 881 [11]. Some studies reported that Y772_A775dup, G776delinsVC, G778-P780dup, and S310F were the most common HER2 mutations in NSCLC [11, 12], of which TP53 was the most frequently co-mutated gene [12]. Many targeted drugs and their corresponding mutation hotspots for HER2 mutations in NSCLC patients have been reported (Fig. 5, Supplementary Table 1). According to the National Comprehensive Cancer Network (NCCN) guidelines, HER2 inhibitors, such as trastuzumab emtansine (T-DM1) and afatinib, are recommended to treat NSCLC.

De Greve et al. reported for the first time the clinical benefit of afatinib-targeted therapy in lung adenocarcinoma patients with HER2 exon 20 mutations [7]. Subsequently, OU et al. reported a case of advanced lung adenocarcinoma harboring V659E/G660R mutation treated with afatinib and obtained PFS for 18 months [13]. Recently, Shan et al. presented a 53-year-old advanced lung adenocarcinoma patient with HER2 exon 20 p.Y772_A775dup mutation and HER2 amplification. After the failures of multiple therapies, including afatinib and T-DM1, pyrotinib treatment displayed rapid and marked clinical remission and achieved a PFS of 7.5 months [14]. Pyrotinib is an irreversible pan-HER receptor tyrosine kinase inhibitor. In the phase II cohort study of 15 NSCLC patients with HER2 mutations, 400 mg pyrotinib resulted in a median PFS (mPFS) of 6.4 months [15]. Additionally, dacomitinib and poziotinib are irreversible tyrosine kinase inhibitors against HER2, EGFR, and HER4. In a phase II clinical trial, the median overall survival of lung cancers with HER2 mutations after starting treatment with dacomitinib was 9 months (95% CI 7–21 months) [16], and the mPFS of poziotinib was 5.6 months in assessable patients with EGFR and HER2 exon 20 mutations [17]. In addition, lapatinib is another TKI that targets HER2 and shows activity in HER2-mutated NSCLC [18].

Trastuzumab is a monoclonal antibody against HER2 that binds to the extracellular domain of the HER2 receptor [19]. Mazières et al. retrospectively identified 65 NSCLC patients carrying a HER2 mutation in 2013 and observed that the disease control rate (DCR) of trastuzumab-based therapy was 93% (*n* = 15), while that of afatinib was 100% (*n* = 3) [4]. Next, a European EUHER2 cohort study conducted by Mazières et al. showed the DCR and PFS of trastuzumab combined with chemotherapy were 75% and 5.1 months, respectively [20]. On the other hand, T-DM1 is a HER2-targeted antibody-drug conjugate. It has been reported that a lung cancer patient with HER2 insertion (p.A775_G776insYVMA) mutation responded rapidly to T-DM1 [21]. In the phase II basket trial, T-DM1 generated a 44% partial response rate and an mPFS of 5 months in patients with advanced HER2-mutant lung cancer after extensive treatment [22].

By reviewing the literature, it is known that insertions of exon 20 are the most common subtype of HER2 mutant lung cancer, but thus far, information about exon 19 of the HER2 gene is still limited. The HER2 p.Asp769Tyr mutation located in the splicing region of exon 19 of the HER2 gene has been identified as a rare mutation in a series of cancers. Some studies have pointed out that this mutation leads to the constitutive activation of HER2 receptor tyrosine kinase and makes it sensitive to HER2-directed antibodies and small molecular tyrosine kinase inhibitors [23]. In our presented case, we reported for the first time the clinical effect of afatinib with anlotinib in the treatment of HER2 p.Asp769Tyr mutation in lung cancer. Before the treatment with afatinib and anlotinib, the patient had received chemotherapy and chemotherapy combined with anlotinib. Nevertheless, the rising rate of tumor markers decreased during anlotinib treatment, and there was no sign of recurrence or metastasis after CT examination. We speculated that anlotinib still played a partial role. Considering these circumstances, it is plausible that the beneficial effects observed in this patient are enhanced by dual HER2 inhibition.

In addition, the unique feature of this patient was the coexistence of HER2 mutation and other genetic alterations. HER2 amplification has been described as a mechanism of acquired resistance to EGFR-TKIs in EGFR-mutant NSCLC tumors [24]. NF1, RB1, and TP53 are multifunctional tumor suppressor genes and play an essential role in the development of tumors. It has been reported that mutations in NF1, RB1, and TP53 were associated with poor outcome among metastatic breast cancers enriched in HR^+^/HER2 ^-^[25]. Among them, TP53 mutations have been confirmed to be related to the process of neovascularization, which promotes tumor growth and metastasis. Therefore, antiangiogenic inhibitors have a therapeutic effect on patients with TP53 mutations [26]. A case report described the favorable response to anlotinib in three patients with advanced NSCLC harboring TP53 mutation [27]. Of course, the above further proved that the patient had a good effect because of the combination of afatinib and anlotinib.

In conclusion, we are the first to report the efficacy of afatinib combined with anlotinib in the treatment of advanced NSCLC with a novel HER2 mutation. Through dual HER2 inhibition, the patient obtained PFS for 10 months. Therefore, exploratory clinical studies can be conducted for lung adenocarcinoma patients harboring HER2 p.Asp769Tyr mutation. Simultaneously, we also summarized the efficacy of the previous literature on targeted therapy of HER2 mutations in lung cancer.

## Supplementary Information

**Additional file 1.**

**Additional file 2.**

**Additional file 3.**

**Additional file 4.**

**Additional file 5.**

## Data Availability

All data and materials are provided in the manuscript and supplementary material.

## Abbreviations

NSCLC: Non-small cell lung cancer
HER: Human epidermal growth factor
TKI: Tyrosine kinase inhibitor
CT: Computed tomography
IHC: Immunohistochemistry
CK: Cytokeratin
TTF-1: Thyroid transcription factor-1
HE: Hematoxylin and eosin
TNM: Tumor node metastasis
MRI: Magnetic resonance imaging
NGS: Next-generation sequencing
PR: Partial response
SD: Stable disease
PD: Progressive disease
PFS: Progression-free survival
MPFS: Median progression-free survival
T-DM1: Trastuzumab emtansine
DCR: Disease control rate
NCCN: National Comprehensive Cancer Network
