# Supplementary figures and images for: Afatinib combined with anlotinib in the treatment of lung adenocarcinoma patient with novel HER2 mutation: a case report and review of the literature
Source: World J Surg Oncol. 2021 Nov 18;19:330. doi: 10.1186/s12957-021-02444-7 (PMC8600784; doi:10.1186/s12957-021-02444-7)

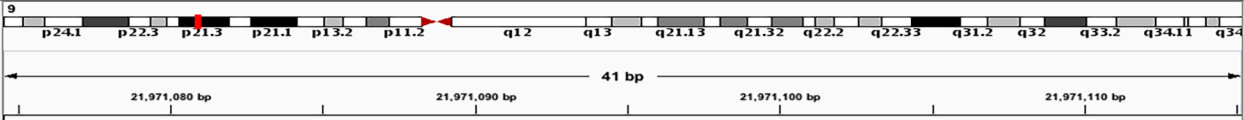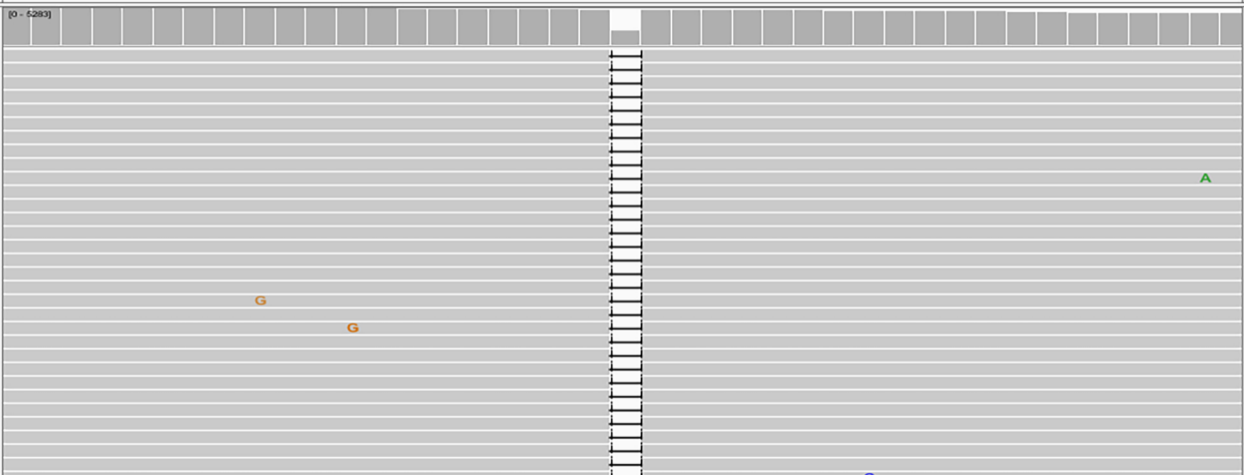

C C A G C G T G T C C A G G A A G C C C T C C C G G G C A G C G T C G T G C A C G  
G A H G P L G G P C R R A R

CDKN2A

Supplement: Supplementary file 1 — Additional file 1. [file 12957_2021_2444_MOESM1_ESM.pdf]

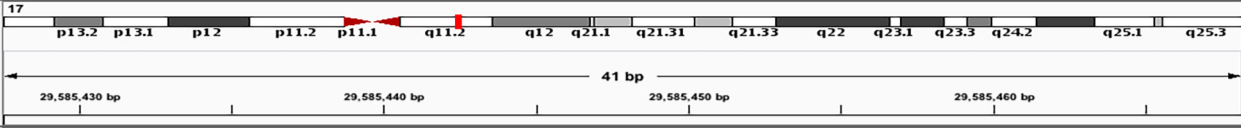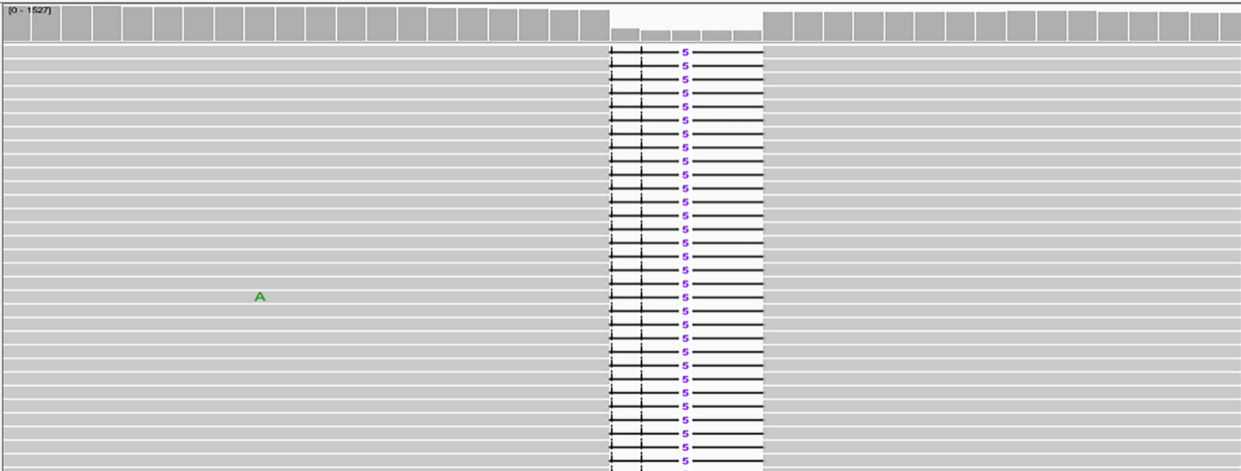

A T C A A T C C T G C C A T T G T C T C A C C G T A T G A A G C A G G G A T T T T

I N P A I V S P Y E A C G I L

Supplement: Supplementary file 2 — Additional file 2. [file 12957_2021_2444_MOESM2_ESM.pdf]

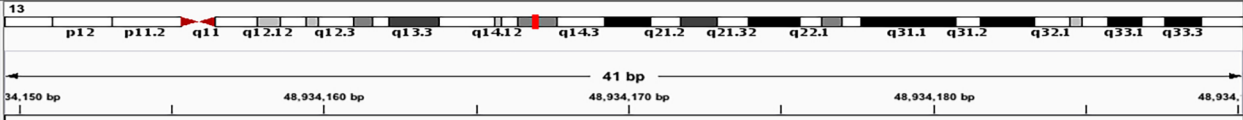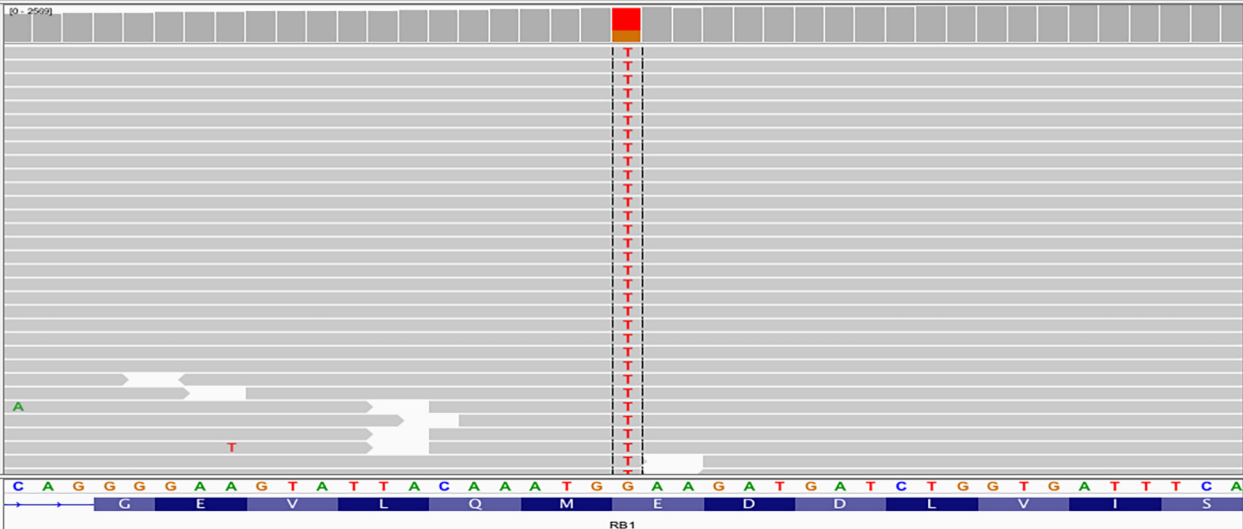

Supplement: Supplementary file 3 — Additional file 3. [file 12957_2021_2444_MOESM3_ESM.pdf]
