# Supplementary material for: Afatinib combined with anlotinib in the treatment of lung adenocarcinoma patient with novel HER2 mutation: a case report and review of the literature
Source: World J Surg Oncol. 2021 Nov 18;19:330. doi: 10.1186/s12957-021-02444-7 (PMC8600784; doi:10.1186/s12957-021-02444-7)
Supplement: Supplementary file 5 — Additional file 5. [file 12957_2021_2444_MOESM5_ESM.docx]

| **Therapy** | **Histological type** | **ERBB2 (HER2) Gene Variant** | **Co-current genes** | **PFS/month** | **Reference** |
| --- | --- | --- | --- | --- | --- |
| **Afatinib** | LUSC | S310Y | EGFR | ＞8.0 | PMID:30584328 |
|  | LUAD | S310Y |  | 5.0 | PMID:29561699 |
|  | LUAD | V659E |  | 5.0 | PMID:27903463 |
|  | NSCLC | V659E | EGFR | <2.0 | PMID:32478891 |
|  | NSCLC | V659E |  | 8.0 | PMID:32478891 |
|  | LUAD | V659E/G660R |  | 18.0 | PMID:27903463 |
|  | LUNG CANCER | G660D |  | >16.0 | PMID:29146616 |
|  | NSCLC | G660D | EGFR+PIK3CA | >4.0 | PMID:32478891 |
|  | LUAD | D769Y |  | 7.0 | PMID:31649001 |
|  | LUAD | Y772_A775dup |  | 3.0 | PMID:22325357 |
|  | LUAD | Y772_A775dup |  | 10.0 | PMID:26559459 |
|  | LUAD | Y772_A775dup | TP53 | 5.0 | PMID:26964772 |
|  | LUAD | G776L | EGFR | 4.0 | PMID:22325357 |
|  | LUAD | G776delinsLC |  | 7.0 | PMID:32075402 |
|  | LUAD | G776delinsVC |  | 12 | PMID:30379401 |
|  | NSCLC | YVMA 776-779 ins |  | 4.0 | PMID:27825109 |
|  | NSCLC | YVMA 776-779 ins | KRAS | 1.0 | PMID:27825109 |
|  | LUAD | V777L | EGFR | >1.0 | PMID:30295016 |
|  | LUAD | G778_P780dup | EGFR | 4.0 | PMID:22325357 |
|  | LUAD | G778_P780dup |  | 3.0 | PMID:26134234 |
|  | LUAD | G778_P780dup | ERBB3 | 9.0 | PMID:31649001 |
|  | LUAD | G778_P780dup | TP53 | 11.0 | PMID:26964772 |
|  | LUAD | G778_P780dup |  | 7.0 | PMID:28363995 |
|  | NSCLC | GSP 781-783 ins | TP53 | 5.5 | PMID:27825109 |
|  | LUAD | N813D |  | 3.0 | PMID:29215816 |
| **Therapy** | Histological type | ERBB2 (HER2) Gene Variant | Co-current genes | PFS/month | Reference |
|  | LUSC | R896G |  | >14 | PMID:31849493 |
| **Pyrotinib** | NSCLC | L755P |  | NA | PMID:30596880 |
|  | LUAD | D769Y/D742N | EGFR | 8.0 | PMID:32327210 |
|  | LUAD | Y772_A775dup |  | 7.5 | PMID:33061462 |
|  | LUAD | Y772_A775dup | TP53 | ＞2.0 | PMID:32458584 |
|  | NSCLC | Y772_A775dup |  | 4.1 | PMID:30596880 |
|  | NSCLC | G776C |  | NA | PMID:30596880 |
|  | NSCLC | G776delinsVC |  | NA | PMID:30596880 |
|  | NSCLC | G778_P780dup |  | ＞12.0 | PMID:30596880 |
|  | LUAD | G778_S779insCPG |  | 12.8 | PMID:32850330 |
|  | LUAD | G780_P781dupGSP |  | 12.5 | PMID:32850330 |
| **Dacomitinib** | LUAD | Y772_A775dup |  | 3.0* | PMID:25899785 |
|  | LUAD | M774delinsWLV |  |  |  |
|  | LUAD | G776delinsVC |  |  |  |
|  | LUAD | G776delinsLC |  |  |  |
|  | LUAD | V777L |  |  |  |
|  | LUAD | G778_P780dup |  |  |  |
| **Trastuzumab** | LUAD | Y772_A775dup | RB1 | 8.0 | PMID:28167203 |
|  | LUNG CANCER | Y772_A775dup | PIK3CA | 5.0 | PMID:28167203 |
|  | LUAD | Y772_A775dup |  | 6.0 | PMID:31649001 |
|  | LUAD | A775_G776 insSVMA |  | 6.0 | PMID:28167203 |
|  | LUAD | G776L | EGFR | >4.0 | PMID:16775247 |
|  | LUAD | G776VinsC |  | 9.0 | PMID:32700047 |
| **Poziotinib** | LUAD | Y772_A775dup |  | 5.5 | PMID:30149884 |
|  | LUAD | G776VinsC |  | 6.0 | PMID:32700047 |
| **Therapy** | Histological type | ERBB2 (HER2) Gene Variant | Co-current genes | PFS/month | Reference |
|  | LUAD | G776delinsVC |  | 4.5 | PMID:30149884 |
| **Lapatinib** | NSCLC | V659E |  | 8.0 | PMID:32478891 |
| **T-DM1 (Trastuzumab emtansine)** | LUAD | Y772_A775dup |  | NA | PMID:25789838 |

**Table 1** Summary of the efficacy of targeted therapy for lung cancers harboring HER2/ERBB2 mutation.

*NA means to have a clinical effect but no specific PFS.

*PFS refers to the mPFS in dacomitinib treatment of HER2 mutations.
