# Supplementary material for: Afatinib combined with anlotinib in the treatment of lung adenocarcinoma patient with novel HER2 mutation: a case report and review of the literature
Source: World J Surg Oncol. 2021 Nov 18;19:330. doi: 10.1186/s12957-021-02444-7 (PMC8600784; doi:10.1186/s12957-021-02444-7)
Supplement: Supplementary file 4 — Additional file 4. [file 12957_2021_2444_MOESM4_ESM.pdf]

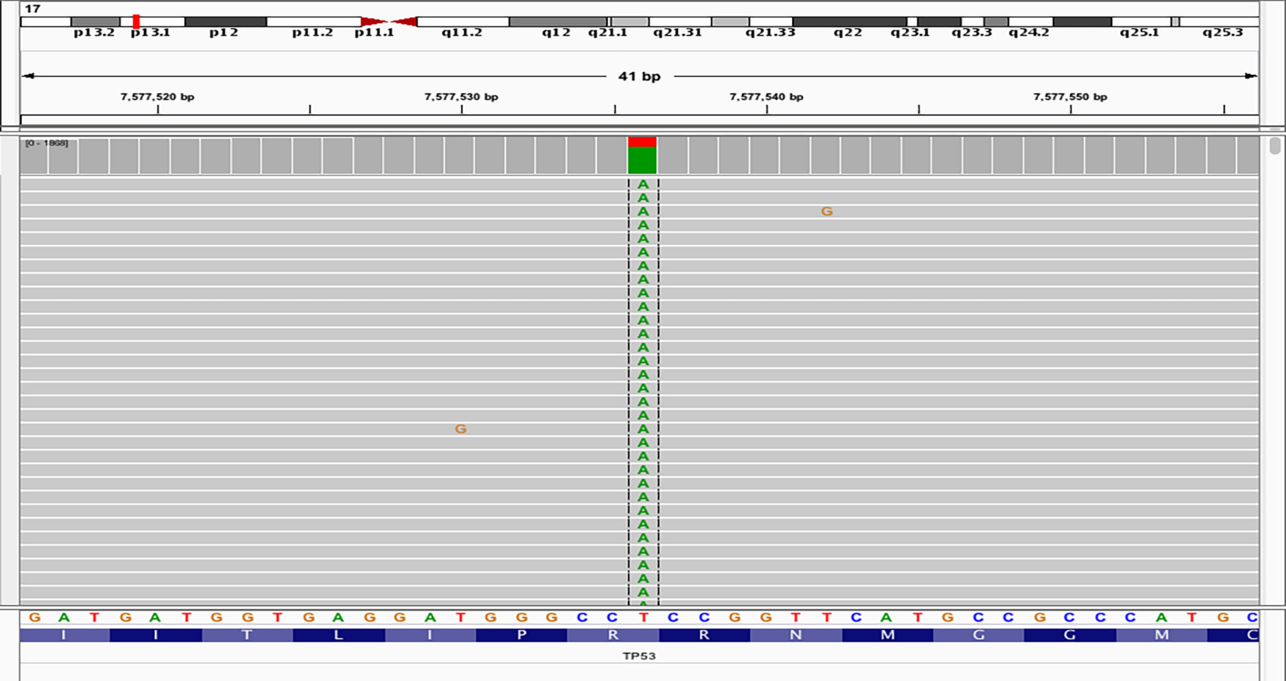
17
p13.2 p13.1 p12 p11.2 p11.1 q11.2 q12 q21.1 q21.31 q21.33 q22 q23.1 q23.3 q24.2 q25.1 q25.3
41 bp
7,577,520 bp
7,577,530 bp
7,577,540 bp
7,577,550 bp
[0 - 1868]
G A T G A T G G T G A G G A T G G G C C T C C G G T T C A T G C C G C C C A T G C
I I T L I P R R N M G G M C
TP53
